# Genetic regulation of diapause and associated traits in *Chilo partellus* (Swinhoe)

**DOI:** 10.1038/s41598-020-58640-0

**Published:** 2020-02-04

**Authors:** Mukesh K. Dhillon, Fazil Hasan, Aditya K. Tanwar, Jagdish Jaba, Naveen Singh, Hari C. Sharma

**Affiliations:** 1 Division of Entomology, ICAR-Indian Agricultural Research Institute, New Delhi, 110012 India; 2 Division of Genetics, ICAR-Indian Agricultural Research Institute, New Delhi, 110012 India; 3 ICRISAT, Patancheru, 502324 Telangana India; 4 Dr. YS Parmar University of Horticulture & Forestry, Nauni, 173230 Solan, Himachal Pradesh India

**Keywords:** Ecology, Genetics

## Abstract

Diapause is an endocrine controlled arrested metabolic state to delay development or reproduction under unfavorable conditions. To gain an understanding of importance of diapause for ecological adaptation, it is important to study regulation of diapause in insects. We examined genetics of diapause in *Chilo partellus* by crossing the hibernating (HD), aestivating (AD), post-hibernating (PHD), post-aestivating (PAD), and nondiapause (ND) strains. Reciprocal crosses were also made to gain full understanding of diapause regulation and the maternal effects, if any. Data were recorded on fecundity, egg hatching, larval survival, diapause induction and termination, adult emergence, and morphometrics of larvae, pupae and adults in the parents (P_1_, P_2_), F_1_ hybrids, and the reciprocal crosses. Genetic analysis showed that AD strain is general combiner, which also improved egg hatching, larval survival, diapause termination, adult emergence and proportion of females in the progenies. Incidence of diapause was highest in HD × AD, whereas termination was greatest in PHD × AD. However, ND strain and its reciprocal crosses with other strains did not exhibit any noticeable developmental response associated with diapause. Specific combining ability analysis suggested that where PHD and AD strains exist together there will be likely reduction in diapause incidence, increased survival with greater fitness and faster multiplication of their progenies resulting in outbreak of *C. partellus*. Degree of dominance estimates revealed that diapause, developmental and morphometric traits in *C. partellus* are governed by over dominance gene effects, and mainly depend on parental diapause history.

## Introduction

Diapause is an endocrine controlled physiological state of arrested metabolic activity during a particular stage of insect development to survive under predictable adverse climatic conditions^1–4^. This happens to control the physiological processes and morphological development during particular stage of life cycle. The insects undergoing diapause pass through a series of physiological events such as suppression of development and reproductive functions, arrested metabolic activity to conserve the reserves, and resumption of normal developmental process on the onset of optimum climatic conditions^3,5–7^. Differences in genetic basis of various components of diapause also vary in different insect species^8^.

Diapause being an adaptive but genetically regulated trait provide phenotypic plasticity to insects in response to environmental conditions^6^. Abiotic factors like cooling and freezing, and rates of temperature change influence developmental and physiological alterations^9–11^, which also lead to several morphological changes such as body color, length, weight and width of various hard sclerotized structures like head capsule, mandible and body appendages^12–15^. Moreover, wider geographic distribution leads to behavioral and physiological differences in populations inhabiting different ecological niches^16^. Existence of ecotypes in different insect species is widely prevalent, and the inheritance of such traits may be due to long-term genetic differentiation or direct physiological response to environmental conditions^17,18^.

Diapause has been reported to follow a simple Mendelian inheritance (3:1 segregation ratio in the F_1_ progenies) in some insect species such as linden bug, *Pyrrhocoris apterus* L^19^., spider mite, *Tetranychus pueraricola* Ehara & Gotoh^20^, and flesh fly, *Sarcophaga bullata* (Parker)^21^. The incidence and duration of diapause in many insect species is under polygenic control^6,22^. Sex-linkage, maternal or paternal effects, and epistasis also play a significant role in diapause regulation in several other insect species^16,23–26^. Genetic and genetic-environmental interaction has been found to be involved in induction and termination of larval diapause in *Ostrinia furnacalis* (Guenée)^27^.

The spotted stem borer, *Chilo partellus* (Swinhoe) is widely distributed in tropical and temperate areas in Asia and Africa, and is a serious pest of maize and sorghum^14,28^. It undergoes facultative diapause as mature larvae inside the stems or stubbles of sorghum and maize^29,30^. Our earlier studies have generated comprehensive information on critical threshold conditions for induction and termination of diapause, duration of diapause, phenology of diapausing larvae, supernumerary moults, effects of diapause on post-diapause development and reproductive physiology, population buildup after diapause, and temperature-based development model of diapausing larvae in *C. partellus*^11,14,15,31,32^. Recent studies although revealed strong gene by environment interaction effects on diapause induction and incidence in some lepidopteran insect species, where inheritance patterns and dominance were found dependent on the photoperiod^27,33,34^. These studies further suggested that the use of varying photoperiod and temperature regimes, and different cross-mating combinations are integral to better understand the inheritance of diapause in insects. In this view our recent studies revealed that both temperature and photoperiod combine are critical for induction and termination of diapause in *C. partellus*^14,15^. Dhillon and Hasan^32^ elaborated that the larvae of *C. partellus* pass through hibernation under North Indian and aestivation under South Indian environmental conditions. The cross-mating among the adults of diapause and nondiapause strains and their F_1_ progenies within and across geographical regions is very likely, and there is a possibility of genetic polymorphism within and/or among the *C. partellus* populations. Thus, to understand such a complex population regulation mechanism, there is an urgent need for intensive genetic research on insects to explore alternative ways to manage insect pests of economic importance. None of the earlier studies attempted such a comprehensive genetic research to understand the genetic regulation of diapause and associated developmental traits in *C. partellus* under different geographical and population scenarios. Therefore, present studies were planned to investigate: i) the genetic components of diapause regulation and developmental traits, ii) effect of parental genetic background on diapause and related traits, and iii) effect of frequency of type(s) of pre-existing strain(s) on diapause incidence, developmental traits, and likely rate of *C. partellus* population buildup under given climatic conditions.

## Results

### Developmental response and incidence of diapause in the diapause and nondiapause parental progenies and the diallel cross populations of *C. partellus*

**Figure 1:**
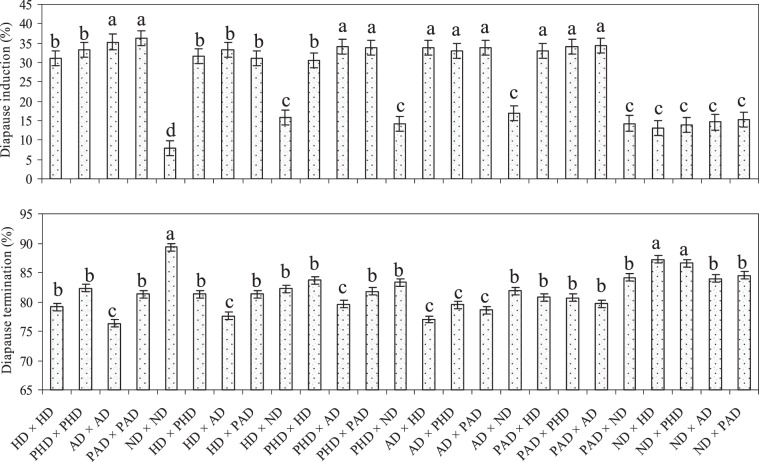
Diapause cycle recorded in the progenies of parents and the intermated populations of *C. partellus*. Diapause incidence (%) out of total larvae survived including diapausing and nondiapausing. Term “Diapause termination” means formation of pupa. (HD = Hibernation population; PHD = Post-hibernation population; AD = Aestivation population; PAD = Post-aestivation population; ND = Nondiapause population). Bars with different letters are significantly different (*Tukey’s HSD*; P > 0.05).

There were significant differences in numbers of eggs laid (F = 12.26; df = 24, 96; P < 0.001), percentage egg hatching (F = 4.77; df = 24, 96; P < 0.001), larval survival (F = 13.28; df = 24, 96; P < 0.001) and adult emergence (F = 13.52; df = 24, 96; P < 0.001) (Table 1) among the parents and the diallel cross progenies of *C. partellus*. Similarly, incidence of diapause (F = 108.15; df = 24, 96; P < 0.001) and diapause termination (F = 13.42; df = 24, 96; P < 0.001) was also significantly different among the parents and the diallel cross progenies (Fig. 1). Among the parental populations, the numbers of eggs/female, percentage egg hatching, larval survival, diapause termination and adult emergence were significantly greater, while diapause incidence was significantly lower in nondiapause strain as compared to the diapause (HD and AD) and post-diapause (PHD and PAD) strains (Table 1). Egg laying by *C. partellus* females, egg hatching and larval survival were also significantly higher in the cases where either of the parent from nondiapause strain was crossed with a parent from diapause or post-diapause strains as compared to other cross combinations, although there were a few exceptions (Table 1). Conversely, incidence of diapause was significantly greater in the larvae obtained from the crosses involving diapause and/or post-diapause strains as compared to the nondiapause strain. However, the diapause termination and adult emergence were comparatively more in the progenies where either of the parent from nondiapause was crossed with a parent from diapause or post-diapause strains as compared to other cross combinations (Table 1; Fig. 1). The number of eggs, egg hatching, larval survival, incidence of diapause, diapause termination and adult emergence were similar in the progenies of the crosses involving diapause and/or post-diapause strains, except in a few cases (Table 1; Fig. 1).

**Table 1:** Mean values of developmental response and diapause incidence recorded in the progenies of parents and the intermated strains of *C. partellus*.

Parents and their diallel crosses	Total pairs observed (No.)	Eggs/ female	Eggs hatching (%)	Larval survival (%)	Adult emergence (%)
**Parents (**♀ × ♂**)**					
HD × HD	59	126.9o	53.3 f	48.3 g	88.0d
PHD × PHD	41	135.8n	57.7d	50.9 f	92.2b
AD × AD	54	159.7j	59.8c	53.4e	83.6e
PAD × PAD	45	208.8e	63.0b	54.4d	87.0d
ND × ND	75	306.2a	78.0a	66.9a	95.0a
**Diallel crosses (**♀ × ♂**) including reciprocals**					
HD × PHD	54	132.8n	53.7 f	49.6 f	89.8d
HD × AD	68	143.5 l	52.3 f	50.2 f	87.8d
HD × PAD	48	150.9k	56.2e	49.5 f	88.0d
HD × ND	62	230.3d	61.4b	49.1 f	91.8c
PHD × HD	50	129.7o	56.0e	51.6	91.5c
PHD × AD	50	139.8 m	58.6d	55.5d	89.2d
PHD × PAD	42	166.5i	53.6e	51.8e	90.6c
PHD × ND	45	248.3b	62.7b	55.1d	94.4a
AD × HD	62	140.2 l	55.2e	51.7e	87.0d
AD × PHD	70	130.5n	61.2b	56.1d	88.5d
AD × PAD	62	182.2 g	60.6c	53.8e	87.0d
AD × ND	61	227.9d	64.3b	62.5a	92.0b
PAD × HD	43	151.6k	58.4d	51.0e	87.0d
PAD × PHD	39	173.5 h	55.9e	52.7e	89.2d
PAD × AD	42	189.5 f	63.9b	49.7 f	88.6d
PAD × ND	63	228.7d	60.0c	59.9c	90.3b
ND × HD	68	244.6c	62.9b	52.7d	93.5a
ND × PHD	44	249.8b	61.8c	57.1c	94.6a
ND × AD	69	231.2d	62.8b	60.8b	92.3b
ND × PAD	48	249.8b	64.2b	61.9b	90.6c
P-value		< 0.001	< 0.001	< 0.001	< 0.001
LSD (P = 0.05)		40.89	6.91	3.74	2.15

The values in a column with different letters are significantly different (Tukey’s HSD; P > 0.05).

### Morphometric differences in various developmental stages of the progenies of diapause and nondiapause parents, and the diallel cross populations of *C. partellus*

**Figure 2:**
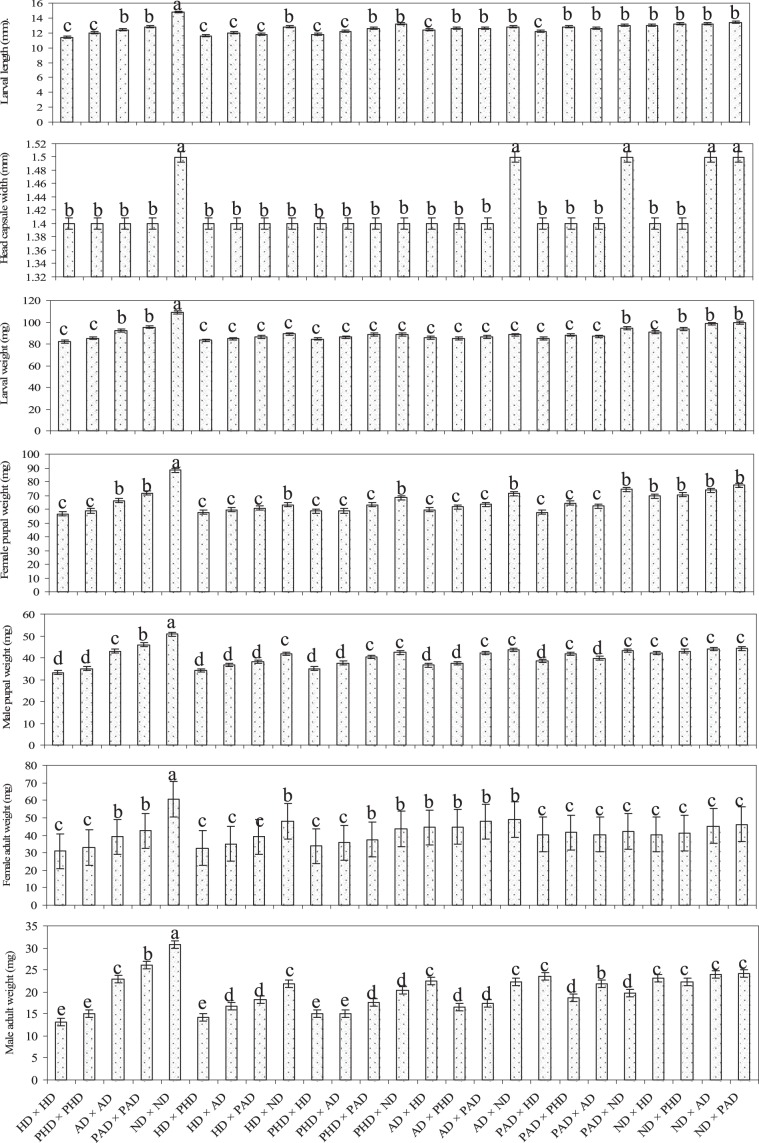
Mean values of morphometric parameters recorded on different developmental stages of diapausing and non-diapausing parents and intermated populations of *C. partellus*. (HD = Hibernation population; PHD = Post-hibernation population; AD = Aestivation population; PAD = Post-aestivation population; ND = Nondiapause population). Bars with different letters are significantly different (*Tukey’s HSD*; P > 0.05).

There were significant differences in larval length (F = 3.13; df = 24, 96; P < 0.001), head capsule width (F = 8.56; df = 24, 96; P < 0.001), larval weight (F = 11.53; df = 24, 96; P < 0.001), female pupal weight (F = 57.75; df = 24, 96; P < 0.001), male pupal weight (F = 16.35; df = 24, 96; P < 0.001), female adult weight (F = 28.56; df = 24, 96; P < 0.001), and male adult weight (F = 16.35; df = 24, 96; P < 0.001) across parents, and the diallel cross progenies of *C. partellus*. The larval length, head capsule width, larval weight, female pupal weight, male pupal weight, female and male adult weights were significantly greater in nondiapause strains as compared to the diapause (HD and AD) and post-diapause [PHD and PAD) parental strains of *C. partellus* (Fig. 2). Furthermore, larval weight, female and male pupal weights, female and male adult weights were significantly greater in the progenies of aestivation and post-aestivation parental strains as compared to hibernation and post-hibernation strains of *C. partellus*. The larval length and head capsule width in the progenies of all the diallel crosses were similar with each other, although there were a few exceptions. Larval, female and male pupal, and female and male adult weights were significantly higher in the cases where either of the parent from nondiapause strain was crossed with another parent from diapause or post-diapause strain as compared to other cross combinations, except in a few cases (Fig. 2).

### Genetic parameters of diapause, developmental and morphometric traits of *C. partellus*

The genetic analysis of incidence and termination of diapause, and adult emergence revealed significantly high variance due to specific combining ability (σ^2^sca) than general combining ability (σ^2^gca) effects (Table 2). The diapause incidence and adult emergence showed higher additive variance than the dominance variance, while the diapause termination showed higher dominance variance than the additive variance (Table 2). Egg hatching and larval survival (Table 2), and morphological traits *viz*., larval weight, and female and male pupal and adult weights (Table 3) also showed high variance due to σ^2^sca effects and dominance variance than the variance due to σ^2^gca and additive variance, respectively. The estimates for narrowsense (h_ns_^2^) and broadsense (h_b_^2^) heritability of diapause and associated developmental and morphometric traits ranged from 0.04 to 0.99 (Tables 2, 3). Incidence and termination of diapause and adult emergence exhibited moderate to high ( > 0.30) h_ns_^2^ (Table 2), while egg hatching, larval survival, larval length, head capsule width, larval weight, and female and male pupal and adult weights, showed very low ( < 0.20) h_ns_^2^ (Tables 2, 3). However, all the diapause and associated developmental and morphometric traits exhibited very high ( > 0.80) h_b_^2^ (Tables 2, 3). The estimates of degree of dominance varied from 1.49 to 30.27 across diapause strains and the associated developmental and morphometric traits (Tables 2, 3).

**Table 2:** Estimates of various genetic parameters for different developmental and diapause traits of *C. partellus*.

Genetic parameters	Egg hatching (%)	Larval survival (%)	Diapause incidence (%)	Diapause termination (%)	Adult emergence (%)
GCA variance (σ^2^gca)	4.02	0.61	24.16	2.02	2.68
SCA variance (σ^2^sca)	34.47	24.58	39.48	7.29	4.43
σ^2^gca/ σ^2^sca	0.12	0.16	0.61	0.28	0.61
Additive variance (σ^2^a)	8.04	1.22	48.32	4.04	5.37
Dominance variance (σ^2^d)	34.47	24.58	39.48	7.30	4.43
Degree of dominance	4.94	7.19	1.49	2.50	1.62
Proportion of dominant and recessive alleles in the parents	0.75	1.26	0.89	0.76	0.73
Proportion of alleles with increasing and decreasing effects	0.24	0.24	0.22	0.24	0.23
Broad sense heritability (h_b_^2^)	0.83	0.92	0.99	0.93	0.94
Narrow sense heritability (h_ns_^2^)	0.16	0.04	0.54	0.33	0.52

**Table 3:** Estimates of various genetic parameters for different morphometric traits of *C. partellus.*

Genetic parameters	Larval length (mm)	Head capsule width (mm)	Larval weight (mg)	Female pupal weight (mg)	Male pupal weight (mg)	Female adult weight (mg)	Male adult weight (mg)
GCA variance (σ^2^gca)	0.07	0.00	2.10	6.13	1.76	7.20	1.76
SCA variance (σ^2^sca)	0.56	0.00	50.96	72.54	26.24	60.09	26.24
σ^2^gca/ σ^2^sca	0.13	0.07	0.04	0.08	0.07	0.12	0.07
Additive variance (σ^2^a)	0.14	0.00	4.19	12.56	3.52	14.41	3.52
Dominance variance (σ^2^d)	0.56	0.00	50.96	72.54	26.24	60.04	26.24
Degree of dominance	30.27	8.95	10.31	4.75	4.79	3.97	4.79
Proportion of dominant and recessive alleles in the parents	1.71	1.77	2.23	1.73	1.79	1.08	1.79
Proportion of alleles with increasing and decreasing effects	0.18	0.20	0.19	0.19	0.20	0.20	0.20
Broad sense heritability (h_b_^2^)	0.84	0.93	0.93	0.99	0.96	0.98	0.96
Narrow sense heritability (h_ns_^2^)	0.17	0.11	0.07	0.14	0.11	0.19	0.11

### General combining ability effects of diapause and nondiapause populations for diapause, developmental and morphometric traits of *C. partellus*

The GCA effects were significant and positive for egg hatching and larval survival in AD, diapause incidence in HD, PAD and ND, and diapause termination and adult emergence in PHD and AD strains (Table 4). However, the GCA effects were significant and negative for egg hatching and larval survival in PAD, diapause incidence in AD, and diapause termination and adult emergence in HD and PAD strains (Table 4). The GCA effects were significant and positive for larval length, head capsule width, larval weight, female and male pupal weights, female and male adult weights in AD, female pupal weight in PHD and female adult weight in ND strains of *C. partellus* (Table 5). However, the GCA effects were significant and negative for larval length, head capsule width, larval weight, female and male pupal weights, female and male adult weights in PAD, female pupal and adult weights in HD, female pupal weight in ND, and female adult weight in PHD strains of *C. partellus* (Table 5).

**Table 4:** General combining ability of diapause experienced and nondiapause parental strains for various developmental and diapause traits of *C. partellus.*

Parental populations and their crosses (×)	Egg hatching (%)	Larval survival (%)	Diapause incidence (%)	Diapause termination (%)	Adult emergence (%)
HD × HD	−0.33	0.06	4.28**	−1.15**	−1.21**
PHD × PHD	−1.21	0.16	−0.39	0.67*	0.60*
AD × AD	3.68**	1.27*	−8.19**	2.19**	2.58**
PAD × PAD	−2.43*	−1.45**	3.25**	−1.38**	−1.57**
ND × ND	0.29	−0.03	1.06**	−0.34	−0.40
Gi-Gj	7.31**	0.65**	2.46***	2.33**	1.91**

*,** = The GCA values significant at P = 0.05 and 0.01, respectively. HD = Hibernation; PHD = Post-hibernation; AD = Aestivation; PAD = Post-aestivation; ND = Nondiapause.

**Table 5:** General Combining ability of diapause experienced and nondiapause parental strains for morphometric parameters of various developmental stages of *C. partellus*.

Parental populations and their crosses (×)	Larval length (mm)	Head capsule width (mm)	Larval weight (mg)	Female pupal weight (mg)	Male pupal weight (mg)	Female adult weight (mg)	Male adult weight (mg)
HD × HD	−0.22	−0.01	−0.46	−1.49**	−0.42	−3.30**	−0.42
PHD × PHD	0.06	0.00	0.80	1.06**	0.06	−0.93*	0.06
AD × AD	0.46**	0.02**	2.40**	3.77**	2.18**	3.95**	2.18**
PAD × PAD	−0.31*	−0.01*	−1.80*	−2.66**	−1.68**	−0.96*	−1.68**
ND × ND	0.01	0.00	−0.94	−0.69*	−0.14	1.24**	−0.14
Gi-Gj	0.92**	0.03**	5.00**	2.45**	2.76**	3.34**	2.76**

*,** = The GCA values significant at P = 0.05 and 0.01, respectively. HD = Hibernation; PHD = Post-hibernation; AD = Aestivation; PAD = Post-aestivation; ND = Nondiapause.

### Specific combining ability effects of diapause experienced and nondiapause populations for diapause, developmental and morphometric traits of *C. partellus*

There were significant and positive SCA effects of PHD female × AD male for egg hatching and larval survival; HD female × AD male, PHD female × ND male and AD female × PAD male for diapause incidence; PHD female × AD male and PAD female × ND male for diapause termination; and PHD female × AD male and PAD female × ND male for adult emergence (Table 6). Great number of PHD and AD strains together resulted into reduced diapause incidence, and increased fitness related traits such as egg hatching, larval survival, diapause termination and adult emergence. Significant and negative SCA effects were found in cases of HD female × PHD male, HD female × AD male, AD female × PAD male and AD female × ND male for larval survival; PHD female × AD male, AD female × ND male and PAD female × ND male for diapause incidence; HD female × PAD male and PHD female × ND male for diapause termination; and HD female × PAD male, PHD female × ND male and AD female × PAD male for adult emergence (Table 6). Furthermore, the SCA effects of HD female × PAD male, HD female × ND male and PHD female × AD male were significant and positive; while that of HD female × PHD male, HD female × AD male, PHD female × PAD male, PHD female × ND male, AD female × PAD male, AD female × ND male and PAD female × ND male were significant and negative for all the test morphometric parameters *viz*., larval length, head capsule width, larval weight, female and pupal weights, and female and male adult weights, except in a few cases where these effects were non-significant (Table 7).

**Table 6:** Specific combining ability of diapause experienced and nondiapause crosses for various developmental and diapause traits of *C. partellus.*

Crosses	Egg hatching (%)	Larval survival (%)	Diapause incidence (%)	Diapause termination (%)	Adult emergence (%)
HD × PHD	−4.07	−4.81**	−1.76	−1.44	−1.01
HD × AD	−4.48	−3.36*	8.08**	0.22	1.24
HD × PAD	3.68	1.90	−1.25	−2.16*	−3.24**
HD × ND	4.18	1.45	1.78	1.73	−1.03
PHD × AD	16.97**	12.59**	−12.66**	5.37**	2.22**
PHD × PAD	−1.49	−2.06	−0.28	0.93	1.14
PHD × ND	−5.67	−2.84	3.55**	−3.81***	−1.98*
AD × PAD	−3.89	−3.24*	7.86**	−0.56	−2.67**
AD × ND	−1.44	−5.12**	−6.14**	−0.72	−0.03
PAD × ND	−0.63	0.09	−2.83**	4.38**	3.79**

*,** = The SCA values significant at P = 0.05 and 0.01, respectively. HD = Hibernation; PHD = Post-hibernation; AD = Aestivation; PAD = Post-aestivation; ND = Nondiapause.

**Table 7:** Specific combining ability of diapause experienced and nondiapause crosses for various morphometric parameters of various developmental stages *C. partellus*.

Crosses	Larval length (mm)	Head capsule width (mm)	Larval weight (mg)	Female pupal weight (mg)	Male pupal weight (mg)	Female adult weight (mg)	Male adult weight (mg)
HD × PHD	−0.87*	−0.05**	−6.68**	−6.71**	−5.67***	−5.02**	−5.67**
HD × AD	−0.67	−0.03**	−5.08*	−7.02**	−5.98**	−7.71**	−5.98**
HD × PAD	0.51	0.03*	6.12**	6.81**	5.88**	3.41*	5.88**
HD × ND	0.59	0.03**	8.47**	10.44**	7.33**	4.61**	7.33**
PHD × AD	1.85**	0.09**	17.47**	20.04**	9.33**	17.52**	9.33**
PHD × PAD	−0.58	−0.02*	−4.13*	−4.13**	−3.41**	−5.56**	−3.41**
PHD × ND	−0.50	−0.02	−3.59	−4.31**	−2.35*	−5.36**	−2.35*
AD × PAD	−0.78*	−0.01	−2.53	−3.85**	−1.52	−3.85**	−1.52
AD × ND	−0.10	−0.00	−0.79	−3.42**	0.53	2.75*	0.53
PAD × ND	−0.32	−0.01	−1.19	−1.59	−2.41*	−6.53**	−2.41*

*,** = The SCA values significant at P = 0.05 and 0.01, respectively. HD = Hibernation; PHD = Post-hibernation; AD = Aestivation; PAD = Post-aestivation; ND = Nondiapause.

**Figure 3:**
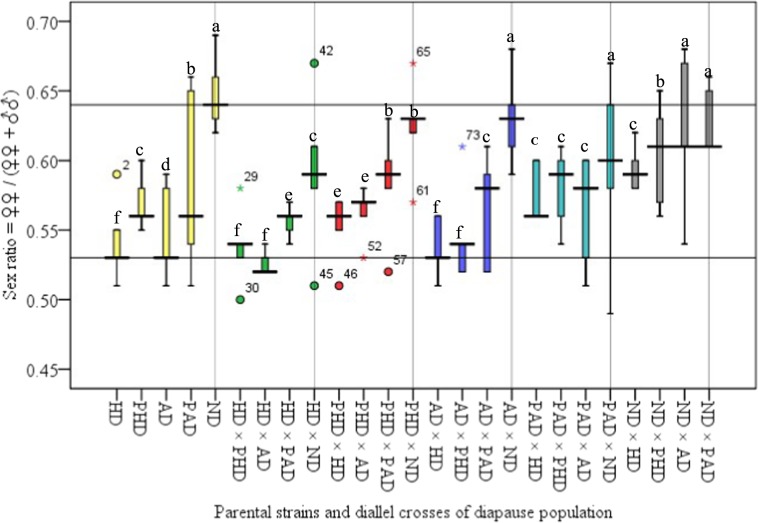
Sex ratio (♀♀/(♀♀ + ♂♂)) of parental strains and diallel crosses of diapause population of *C. partellus*. The limits of a box denote the upper and lower quartiles, the horizontal bar is the median, and the 1.5 Interquartile Ratio (IQR) criteria has been used to classify outliers. (F = 3.81, df = 24, 96, P < 0.001; LSD = 0.18). Box plots with different letters are significantly different (*Tukey’s HSD*; P > 0.05).

The present studies found a female biased sex-ratio in parental strains and their diallel crosses (Fig. 3). The proportion of females was significantly greater in ND and lower in HD as compared to other strains (F = 3.81; df = 24,96; P < 0.001). The sex-ratio in other parental strains and their diallel crosses varied between ND and HD strains (Fig. 3).

## Discussion

Diapause has evolved independently in various insect species, encompassing different life styles to adapt their life cycles to the seasonal changes^35,36^. Greater incidence of diapause in the progenies of diapause and post-diapause strain and their diallel crosses than those from the nondiapause strain in the present studies suggest that the mating between diapause strain greatly increase the propensity of diapause in the progenies. The sex-linked effects for induction of diapause have also been reported in many insect species due to influence of either or both the diapause parents as observed in the present studies^16,25,37–40^. Further, the greater fecundity, egg hatching, larval survival, termination of diapause and adult emergence in the progenies of nondiapause strain and the crosses involving either of the parent from nondiapause in comparison to diapause and post-diapause strains and their crosses suggest that the parental experience play an important role in inheritance of diapause in the progenies. Earlier reports of 15 to 65% diapause in *C. partellus* under different temperature and photoperiod conditions in the laboratory shows that apart from parental diapause history, asynchrony in diapause inducing stage and environmental conditions, and genetic segregation could also be influencing the induction of diapause in the progenies^14,15^. Diapause in *C. partellus* has also been found to affect certain morphological features like larval weight, length, and head capsule width^9,10,14,32^. Present studies revealed that the lower larval weight, shorter length and head capsule width in the progenies of diapause and post-diapause parental strains and their crosses lead to lower weight pupae and adults as compared to nondiapause counterparts (Fig. 2). Small size of the insects in the diapause strain could be because of utilization of body reserves to prepare for diapause^12,13^. Interestingly, it is to be noted that larval weight, female and male pupal weights, female and male adult weights were significantly greater in aestivation experienced strain as compared to hibernation experienced strain of *C. partellus*. It might be due the fact that larvae of *C. partellus* exhibited a prolonged duration during hibernation as compared to aestivation, which resulted into considerable reduction of weight in hibernating experienced strain as compared to aestivating experienced strains. Further, reduction of larval weight in diapausing insects also depends on the duration of diapause, the prolonged the duration of diapause the greater reduction of body weight due to utilization of body reserve during diapause^12,13^.

Significant and positive GCA effects of the *C. partellus* from HD and PAD strains for incidence of diapause suggested that it is genetically controlled and the parental experience play an important role in diapausing behavior (Table 4). Incidence of diapause in *C. partellus* was found to be influenced by both the parents, and the F_1_ progenies exhibited intermediate response as in other insect species^38,41,42^. Moreover, cross-mating of strains from different geographical areas may also result in genetic polymorphism within insect populations for various diapausing traits. Varying diapause responses have also been reported in the progenies of crosses between different geographic and laboratory strains^43,44^, and genetic studies related to diapause have shown different modes of inheritance^6,21,45,46^.

In present studies, the significant and positive SCA effects in certain crosses for various morphometric parameters indicated the involvement of at least one parent from AD and/or HD strain for induction of diapause (Table 7). These findings suggest that if the frequency of PAD and ND type of mixed strains is higher, there are chances of increased fitness in their progenies resulting in built up of the active population at a faster rate. However, the pre-existence of PHD and ND or AD and PAD type of strains under unfavorable climatic conditions could result in drastic reduction in the active population, since such combinations were found to result in increased frequency of diapause incidence in *C. partellus* (Fig. 1). The results further indicate that under the situations where PHD and AD strains exist together there will be reduced diapause induction, increased survival with greater fitness, and faster multiplication of their progenies resulting in outbreak of *C. partellus*. Female biased sex ratio suggested the involvement of either of the parent from nondiapause. Sexual differences in diapause propensity might be due to effect of either of the parents, as males and females are quite dissimilar in life history traits^47^. Generally, the insect females have longer development time than the males^48^, which profits females growing into larger body size and ultimately influences fecundity^49^.

Polygenic inheritance of diapause characteristics has been reported in many insect species, wherein hybrids have often been found intermediate between the parents^45^. Inheritance of diapause induction in European corn borer, *O. nubilalis* has earlier been reported to be regulated by multigenetic, sex-linked, and lack of dominance^24,50,51^. In present studies, the large variance due to SCA than GCA for diapause incidence and adult emergence indicated dominance gene action, while greater variance due to additive than dominance gene effects suggested the involvement of genetic and G × E interactions for these traits (Table 2). Greater SCA variance and dominance gene effects for diapause termination, egg hatching, larval survival and weight, and pupal and adult weights of both the sexes suggested that these traits are controlled by dominance gene action. High broadsense heritability of diapause and associated developmental and morphometric traits, and moderate narrowsense heritability of diapause incidence, termination and adult emergence indicate uniform breeding under laboratory conditions, while the estimated heritability under natural conditions could be over-estimated. Heritability in the laboratory reared strains provides a reasonable estimate in terms of magnitude and significance^52,53^. Heritability estimates for diapause intensity vary from low to very high, depending upon insect species, type of insect population, and experimental conditions^54–59^. Very high heritability estimates for various life history traits could be because of strong natural selection for the traits relevant to fitness, which under diapausing conditions tend to reduce additive genetic variance^60–63^. Further, the degree of dominance estimates for diapause, developmental and morphometric traits revealed that these traits are governed by overdominance gene effects in *C. partellus*, wherein the genetic variability and diapause incidence could be prerequisite for rapid adaptation to prevailing environmental conditions. These studies have implications for exploring appropriate genetic means of *C. partellus* management under different agro-ecological conditions.

## Materials and Methods

### Cultures of *Chilopartellus*

The *C. partellus* larvae were collected from northern Indian region, New Delhi (28°3823’N, 77°0927’E; AMSL 228.61 m) for hibernation population; southern Indian region, Hyderabad (17.3850°N, 78.4867°E; AMSL: 505 m) for aestivation population; and reared on maize stalks till pupation in the insectary at Division of Entomology, ICAR-Indian Agricultural Research Institute, New Delhi under 27 ± 1 °C, 70 ± 5% RH and 12 L:12D conditions. The adults obtained from these cultures were paired in oviposition cages and provided with water soaked in a cotton swab. The oviposition cages were wrapped with a wax-paper on the outer surface to serve as oviposition substrate. The wax-papers were changed daily. The wax-papers with eggs were kept at 27 ± 1 °C, 70 ± 5% RH and 12 L:12D h for egg hatching. Freshly hatched *C. partellus* larvae were inoculated in a plastic container (1000 ml capacity) containing artificial diet^64^, and kept in walk-in insect growth chambers (RINAC Make, 1.83 m L × 1.83 m W × 2.44 m H) at 27 ± 1 °C, 70 ± 5% RH and 12 L:12D). The culture of *C. partellus* was initially obtained from North and South India were pooled and maintained year-round in the insectory at Division of Entomology, ICAR-Indian Agricultural Research Institute, New Delhi, and used as nondiapause strain.

### Formation of different diapause and nondiapause *C. partellus* populations

The late 4^th^ to early 5^th^ instar larvae of *C. partellus* from the above described respective cultures were exposed to earlier standardized hibernation (10 ± 1 °C + 10 L:14D) and aestivation (32 ± 1 °C + 13 L:11D) inducing conditions along with dry diet^14,15^. As a result of diapause treatment, the larvae showing any kind of diapause symptoms described by Dhillon and Hasan^32^ were considered to be in diapause. In general, the dormancy duration in *C. partellus* varies from 45 to 50 days in different agro-ecosystems^14,15^. The diapausing larvae thus obtained were kept undisturbed at the respective treatment conditions up to 45 days, and afterwards exposed to diapause termination conditions (fresh artificial diet; 27 ± 1 °C, 70 ± 5% RH and 12 L:12D conditions). Three batches of *C. partellus* larvae were exposed to hibernation and aestivation inducing conditions at three-week intervals so that the adults obtained from these diapause strains could be picked for mating in different cross combinations. The afore-mentioned year-round laboratory-maintained *C. partellus* culture was used as a nondiapause strain. In one set, the adults obtained from the hibernating, aestivating and nondiapausing larvae were designated as hibernation (HD), aestivation (AD) and nondiapause (ND) strains. In another set, the progenies obtained from the hibernation and aestivation strains were reared under laboratory conditions at 27 ± 1 °C, 70 ± 5% RH and 12 L:12D till adult emergence, and the adults thus obtained were delineated as post-hibernation (PHD) and post-aestivation (PAD) strains, respectively. In this way, there were five parental strains, i.e., HD, AD, PHD, PAD and ND.

### Mating scheme

**Figure 4:**
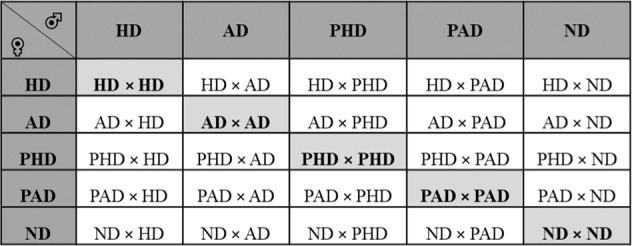
Mating scheme of different diapause and nondiapause *C. partellus* parental populations in all possible combinations including reciprocals in a diallel fashion. The abbreviations used in the mating scheme are elaborated as: HD = Hibernation, AD = Aestivation, PHD = Post-hibernation, PAD = Post-aestivation, and ND = Nondiapause. The diagonal cells marked in light grey color are parental crosses.

The simultaneous existence of different strains *viz*., hibernation, aestivation, nondiapause and their progenies is very likely, and the cross-mating among the adults of these strains within and across geographical regions could result in genetic polymorphism in *C. partellus* populations. Thus, present investigations on genetic components of diapause involved hibernation (HD), aestivation (AD), post-hibernation (PHD), post-aestivation (PAD) and nondiapause (ND) strains of *C. partellus*. The adults of these five parental strains were used in making all possible crosses including reciprocals in a diallel fashion (Fig. 4). The virgin females and males were paired individually in the oviposition cages as per the mating scheme described in Fig. 4. A total of 75 adult pairs were produced for each parental population and their crosses.

### Experimental conditions and observations recorded

The aforesaid 75 male and female pairs of each parental and the diallel crosses were placed such that there was a cohort of 15 pairs per replication, and there were five replications in a completely randomized design (CRD) for each cross combination. The oviposition cages were kept in walk-in insect growth chamber at 27 ± 1 °C, 70 ± 5% RH and 12 L:12D for mating and oviposition. Wax-paper was wrapped around the oviposition cages to serve as oviposition substrate. The wax-papers were changed daily. Eggs laid by each female from each cross combination were recorded daily till the females died. The pairs which did not lay eggs were not included in the experiment. Total numbers of eggs laid by the females in a cohort were divided by the number of egg laying females in each replication, and the data were expressed as number of eggs per female for each mating combination. The wax-papers having egg masses were kept in a plastic bucket having water at the bottom to maintain humidity till the black head stage. At black head stage, the eggs were removed from the bucket and kept in plastic jars for egg hatching. Percentage of egg hatching was recorded for each replication separately. All the neonate larvae from each replication were inoculated on artificial diet^64^ poured in plastic containers (1000 ml capacity), and kept at 27 ± 1 °C and 12 L:12D. After 30 days, five randomly selected *C. partellus* larvae displaying diapause symptoms were removed from each replication, and data were recorded on larval weight, length and head capsule width, and data averaged for each replication. Observations on larval length and head capsule width were recorded using Leica StereoZoom Microscope (Leica Microsystems Ltd, Switzerland), and weight on an electronic balance (Precision balance, CB-Series, Contech). Thereafter, the larvae were placed back in respective jars.

After 45 days, the numbers of larvae transformed into pupae, and the larvae showing diapause symptoms or entered diapause were recorded for each replication in each cross. Larval survival was expressed as numbers of larvae (transformed into pupae + the larvae displaying symptoms or entered diapause) survived/numbers of larvae released × 100. Diapause incidence was calculated as numbers of larvae entering diapause/numbers of larvae released × 100. The numbers of diapause larvae molted into pupae were counted and expressed as diapause termination (%): Numbers of pupae from diapause larvae/numbers of diapause larvae × 100. The number of adults emerged from diapause larvae were also counted and expressed as a percentage of adults emerged from diapause larvae/total number of diapause larvae × 100. The weights of five randomly selected female and male pupae, and the female and male moths emerging from the diapause larvae in each cross combination were measured on an electronic balance (Contech, CB-120) after 24 h of pupation, and at adult death, respectively. The data were averaged for each replication, and expressed as mg/pupa or adult.

### Statistical analysis

Mean values of all the test parameters of progenies of parents and the diallel crosses were used for statistical analysis. Sex ratio was calculated according to Wilson and Hardy^65^ formula [Sex ratio = ♀♀ / (♀♀ + ♂♂)] for each diallel cross, and expressed as proportion of offsprings that were females. Data on developmental parameters, morphometrics during different developmental stages, and sex ratio in the progenies of parents and the diallel cross populations were subjected to one-way analysis of variance (ANOVA) followed by Tukey–Kramer honestly significant difference (HSD) test using SPSS v. 15.0 statistical package (SPSS Inc. Chicago, IL, US). Analysis of variance, component analysis for estimation of degree of dominance, proportion of dominant and recessive alleles, proportion of alleles with positive and negative effects, heritability, and combining ability analysis were performed as per the method suggested by Hayman^66,67^ using Windostat Version 9.2.
